# Association between blood pressure measures and recurrent headache in adolescents: cross-sectional data from the HUNT-Youth study

**DOI:** 10.1007/s10194-011-0304-x

**Published:** 2011-02-08

**Authors:** Erling Tronvik, John-Anker Zwart, Knut Hagen, Grete Dyb, Turid Lingaas Holmen, Lars Jacob Stovner

**Affiliations:** 1 Department of Neurology, Norwegian National Headache Centre, Trondheim University Hospital, 7006 Trondheim, Norway; 2 Department of Neurosciences, Norwegian University of Science and Technology, 7006 Trondheim, Norway; 3 Department of Public Health and General Practice, HUNT Research Centre, Norwegian University of Science and Technology, Trondheim, Norway; 4 Department of Neurology, Ullevål University Hospital, Oslo, Norway; 5 Faculty of Medicine, University of Oslo, Oslo, Norway

**Keywords:** Headache, Migraine, Blood pressure, Epidemiology, Adolescent

## Abstract

The relationship between blood pressure and headache in youth has not been explored and the objective of the present study was to provide data on this association in an adolescent population. Cross-sectional data from a large population-based survey, the Young-HUNT study, on 5,847 adolescents were used to evaluate the association between blood pressure (systolic, diastolic, mean arterial and pulse pressure) and recurrent headache, including migraine and tension-type headache. Increasing pulse pressure was inversely related to recurrent headache prevalence, and both tension-type headache and migraine. For systolic blood pressure such an inverse relationship was present for recurrent headache and tension-type headache prevalence. For migraine, the results were not significant, although there was a tendency in the same direction (*p* = 0.05). High-pulse pressure has previously been found to be inversely related to the prevalence of migraine and tension-type headache in an adult population. This inverse relationship has now been demonstrated to be present among adolescents also, supporting the results from a previous study in adults, that blood pressure regulation may be linked to the pathophysiology of headache.

## Introduction

A relationship between migraine and blood pressure (BP) has long been suspected, but the direction of the association has been controversial [1]. For many years, it was assumed that hypertension might be a cause of headache. Studies supporting this connection were published and the pulsatile characteristics of some headaches and the introduction of β-blockers in migraine prophylactic treatment may further have supported this view [2, 3]. There is, however, a possibility that this observation may be an example of admission rate bias (Berkdon’s bias) as hypertension and headache are common medical disorders and consulting the doctor for one of the disorders may give a higher likelihood of detecting the other condition. Later, epidemiological studies showing no relationship between headache and high-BP were published [4, 5], and in the International Classification of headache disorders II, it is stated that mildly or moderately elevated BP is not a cause of headache [6]. Recent epidemiological data in adult populations suggest even an inverse relationship between headache prevalence and both pulse pressure (PP), and systolic blood pressure (SBP) [7–10]. A little is known about this relation in the younger part of the population, in which, BP values normally are well within the normotensive range. Headache in adolescents is a frequent symptom [11], and the objective of this study was to explore the relation between BP and recurrent headache, including migraine or tension-type headache (TTH) in a large population-based study.

## Materials and methods

The Nord-Trøndelag Health Study (HUNT) is one of the largest epidemiologic studies ever performed. Nord-Trøndelag County is located in the middle of Norway and is fairly representative of the population as a whole and the study is aimed at several large public health issues.

In 1995–1997, all students in junior high schools (aged 13–15 years) and high schools (aged 16–19 years), of the county of Nord-Trøndelag, were invited to participate in the youth part of the Nord-Trøndelag Health Study, the Young-HUNT study. A total of 8,984 students (88%) out of 10,202 invited individuals completed a comprehensive self-administered questionnaire with different health-related items during one school hour. The study followed the school year instead of the calendar year. As adolescents usually graduate from high school, in the calendar year they become 19, about half of the 19-year olds had left school and were not invited. Accordingly, the 19-year-old students in the study population were excluded (*n* = 382). The few 12-year-old students in the first year of junior high were also excluded (*n* = 126). Questionnaires, where the headache question had not been answered (*n* = 221) were excluded, leaving a total study population of 8,255 (81%). Within a month after completing the questionnaire, a clinical examination was performed in schools during school hours and included spirometry, weight, height and BP measurements. The intention was to let the whole population undergo a headache interview performed by trained nurses in connection with the clinical examination [12], but the interviews did not commence until February 1996 and this resulted in a lower number of interviewed individuals. Among the 5,847 adolescents 13–18 years of age that underwent the interview all had valid questionnaires and of these 5,832 had valid BP measurements. There was no major difference in sex and age distribution between the total questionnaire-based study population (*n* = 8,255) and the questionnaire-based population that also were interviewed (*n* = 5,847) [12]. The first question of the interview was whether the students had experienced recurring headaches not related to cold, fever or any other type of illness, and whether they had experienced such headaches during the last 12 months. The students were then asked to classify their headache(s) according to two descriptions [13], one for migraine and the other for TTH (see "Appendix"). These descriptions were based on the International Headache Society (IHS) criteria [14] with the exceptions that the number of attacks and the duration of headaches were not included [15]. The students were also given a third option (non-classifiable headache), in case none of the two descriptions resembled their symptoms. Headache frequency was also registered, and they were asked to indicate the average number of days they had experienced headaches in the past year; less than one day per month (less than monthly), 1–3 days per month (monthly), 1–5 days per week (weekly), more than 5 days per week (daily). The “recognition-based” headache diagnosis obtained by the nurses has been validated previously against extensive semi-structured interviews by neurologists [15]. The positive predictive value for migraine was 89% and the negative predictive value 90%. For TTH, the positive and negative predictive values were 83 and 91%, respectively. The one-year prevalence of migraine was 7%, of TTH 18% and of non-classifiable headache 4.8%. Headache at the time of investigation was not registered. Background data on demographics and headache diagnostic groups and frequency are given in Tables 1, 2 and 3. For more detailed information, see Ref. [12].

**Table 1:** Study population

Schools	Number invited (aged 12–19 years)	Participants with valid questionnaires (aged 13–18 years)			Participants included with valid questionnaires, interviews and blood pressure data (aged 13–18 years)		
		*n*	Mean age (SD)	% Girls	*n*	Mean age (SD)	% Girls
Junior high school	5,004	4,508	14.6 (0.9)	50.5	3,265	14.7 (0.9)	51.0
High school	4,913	3,733	17.4 (0.9)	49.2	2,555	17.5 (0.9)	52.9
‘Not in school’	285	14	17.2 (0.9)	64.3	12	17.2 (0.9)	75.0
Total	10,202	8,255	15.9 (1.7)	49.9	5,832	5.9 (1.6)	51.9

*SD* standard deviation

See Ref. [12] for more detailed information

**Table 2:** The prevalence of overall recurrent headache, and subgroups migraine, tension-type headache, and non-classifiable headache

	Overall recurrent headache (%)	Migraine (%)	Tension-type headache (%)	Non-classifiable headache (%)
Boys *n* = 2,801	581 (20.7)	115 (4.1)	330 (11.8)	117 (4.2)
Girls *n* = 3,031	1,091 (36.0)	226 (7.5)	652 (21.5)	163 (5.4)

Sixty-nine participants had co-occurrence of migraine and tension-type headache and these were excluded from the migraine and tension-type headache groups

**Table 3:** Recurrent headache frequency

	<1 day per month (%)	1–3 days per month (%)	1–5 days per week (%)	>5 days per week (%)
Boys *n* = 2,801	147 (5.2)	279 (10.0)	112 (4.0)	5 (0.2)
Girls *n* = 3,031	174 (5.7)	542 (17.9)	329 (10.9)	24 (0.8)

Blood pressure is a measure of the force applied to the wall of the arteries as the heart pumps blood through the body. The pressure is determined by the force and amount of blood pumped and the size and flexibility of the arteries, and is usually represented by two values. The systolic arterial pressure is defined as the peak pressure in the arteries, which occurs at the beginning of the cardiac cycle. The diastolic arterial pressure is the lowest pressure which is present in the resting phase of the cardiac cycle. BP is also characterized by its pulsatile and steady components. The pulsatile component, estimated by PP (SBP−DBP), represents BP variation during the cardiac cycle and is affected by left ventricular ejection fraction, arterial stiffness, early pulse wave reduction and heart rate. The steady component, estimated by MAP, is a function of left ventricular contractility, heart rate and vascular resistance and elasticity averaged over time [16].

BP was measured with the adolescent sitting [17]. First, the upper arm circumference was measured to the closest centimeter. If the circumference was less than 24 cm, a small cuff was used, if it was between 24 and 34 cm a medium cuff was used, and if it was 35 cm or more, a large cuff was used. The measurements were done using an automatic oscillometric technique (507 N monitor, Criticare System Inc., USA). The adolescents rested for 2 min before the first measurement, and the second and third measurements were done with 1 min intervals. Blood pressure was measured manually when an automatic recording could not be obtained. The mean value of the last two measurements was used in the analyses.

### Ethics

A written consent from the parents was required for students younger than 16 years of age. The Young-HUNT study was approved by the Regional committee for ethics in medical research, and by the Norwegian data inspectorate.

### Statistical analysis

The prevalence odds ratio (OR) for the association between recurrent headache (including subgroups migraine and TTH) and different BP groups was estimated using multiple logistic regression. In order to establish uniform groups, subjects with two or more recurring headaches (i.e., co-occurring migraine and TTH) were omitted from the analyses. The term “recurrent headache” in this paper refers to the headache variable obtained during the interview and includes subjects with migraine, TTH and non-classifiable headache, while “headache” is used as a more general and unspecific term. SBP, diastolic blood pressure (DBP), mean arterial pressure (MAP) and PP were each divided into six categories using the 10, 25, 50, 75 and 90% in the cross-sectional data. Potential confounding was evaluated by adjusting for age and weight. As the results were not affected when weight was added as covariates to the model, this variable was omitted. Due to several statistical calculations, the level of significance was set at *p* < 0.001. A two-sided trend test was performed to evaluate the probability of a linear relationship between BP categories and headache prevalence. To further assess deviation from the linearity assumption, a trend test for BP as a continuous variable was performed for the different headache categories, in which, the trend test for BP as a categorical variable had shown significance. As a continuous variable the *p* value ranged from *p* = 0.002 to *p* < 0.0001. Spearman’s correlation coefficient (*r*_s_) was used to investigate the relationship between headache frequency and BP categories. Statistical analyses were performed using SPSS version 14.0 for Windows (SPSS Inc., Chicago, USA).

## Results

### Systolic and diastolic blood pressure

**Fig. 1:**
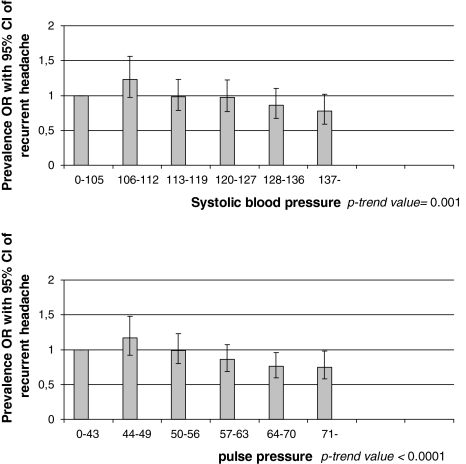
The relation between blood pressure and recurrent headache in both genders combined (ORs with 95% CIs, *n* = 5,832)

There was a linear trend of decreasing prevalence of overall recurrent headache (Fig. 1) and TTH (*p* trend value <0.001) with increasing SBP values analyzing boys and girls together. For migrainous headache the same tendency was observed, but the finding was not significant (*p* = 0.05).

For DBP, there was no association with the prevalence of overall recurrent headache (*p* trend value is 0.36), migraine (*p* trend value is 0.19) or TTH (*p* trend value is 0.64).

When stratifying for sex and diagnostic group, the results were not significant (data not shown).

### Pulse pressure and mean arterial pressure

In boys and girls, combined analyses revealed a highly significant inverse relationship between increasing PP and recurrent headache (Fig. 1), and for migraine (*p* trend value <0.001) and TTH (*p* trend value <0.0001). Stratifying for sex and diagnostic group, this inverse relationship was not significant (data not shown).

**Fig. 2:**
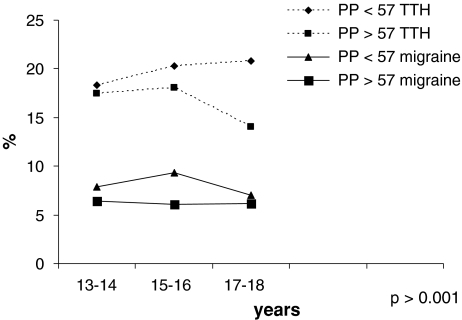
The prevalence of tension-type headache (TTH) and migraine in groups with pulse pressure (PP) above or below the median related to age (both genders)

PP increases with age in the general population. To evaluate the influence of age on the relationship between PP and headache prevalence, we split the data in two, using the median PP value as cut-off, and plotted the prevalence of migraine and TTH versus age (Fig. 2). In both the migraine and the TTH group, there was a tendency for high-PP to be associated with a lower prevalence of headache, but this was not significant.

Mean arterial pressure was not associated with the prevalence of recurrent headache (*p* trend value is 0.37), migraine (*p* trend value is 0.78) or TTH (*p* trend value is 0.07) in the combined analyses or the analyses stratified by gender and diagnostic group (data not shown).

### Blood pressure and headache frequency

Recurrent headache frequency for both genders combined was categorized into four different groups; less than one day per month (less than monthly), 1–3 days per month (monthly), 1–5 days per week (weekly), more than 5 days per week (daily). There was a weak negative correlation between recurrent headache frequency and PP (*r*_s_ = −0.06, *p* < 0.001). For SBP, DBP and MAP, no significant association with recurrent headache frequency was observed.

### Blood pressure and use of pain-relieving medication

To determine whether the level of BP influenced use of pain-relieving medication, use of common analgesics and migraine-specific medication for both genders combined were subclassified into three categories each; never used, periodic use and almost daily use. A weak negative association was present between the use of migraine-specific medication and PP (*r*_s_ = −0.06, *p* < 0.001). For SBP, DBP and MAP, no significant association with use of migraine-specific medication was found. Use of common analgesics showed a weak negative association with PP (*r*_s_ = −0.07, *p* < 0.001) and SBP (*r*_s_ = −0.06, *p* < 0.001). For DBP and MAP, no significant association was observed.

## Discussion

This is a population-based study to investigate a possible association between BP and headache prevalence among adolescents, testing for linear trends. The main finding was the inverse relationship between PP and recurrent headache prevalence, including the subgroups TTH and migraine for boys and girls combined.

The relationship between recurrent headache frequency and PP was weak, but the negative association is the one which would be expected from the direction of the associations between headache prevalence and the BP variables.

Similarly, the fact that use of migraine-specific medication and common analgesics was lower in those with higher PP and supports the main finding of a negative association between PP and recurrent headache.

Results from this adolescent population is in accordance with data from an adult population from the HUNT study, in which high-systolic and high-pulse pressure were associated with less migraine and non-migrainous headache [7]. Similar results have been published by other groups [8–10, 18, 19]. The inverse relationship is not restricted to headache, but seems to be valid for pain in other parts of the body as well [20]. The findings seem to be more consistent for headache in general than for migraine [7], and the present study demonstrates that this inverse relationship is present even in adolescents, who in general are normotensive.

We have previously argued that [7], most likely, this inverse relationship between BP and headache prevalence is the result of a phenomenon called hypertension associated hypalgesia [21, 22]. There is evidence that a higher level of BP may inhibit pain transmission through stimulation of the baroreflex system, possibly due to an interaction between nociceptive and cardiovascular centers in the brainstem [22]. This phenomenon is also present within the normotensive range [21], a fact that is in accordance with the results presented in this study.

Strengths of the study are the large and unselected population and the use of validated diagnoses. A limitation of this study is that only cross-sectional data are available. Another limitation is that the results did not remain significant with stratification for gender, and as there was no interaction by gender, this was probably due to lack of power. The relatively narrow range of BP-values in an adolescent population may also explain the need for a large number of subjects in the analyses.

The high participation rate in the questionnaire-based part of the study indicates that the study cohort represents the population fairy well, and that no serious selection bias was present. It also seems reasonable to assume that the prevalence rates of recurrent headaches based on data from the interviewed population are fairly representative. There were no systematic selection of subjects and no major differences in age and sex distribution between the total questionnaire-based study population and questionnaire-based study population that also were interviewed [12]. There is probably a little or no interest-related selection bias, since headache and BP were only a few among many other objects of the study. A total of 4.8% of the interviewed population reported their headache not to be consistent with either migraine or TTH and were categorized as non-classifiable headache. Others have reported higher rates of non-classifiable headaches among adolescents [23, 24], and the applicability of the IHS criteria in the pediatric population has been debated. The recognition-based technique has been validated but may underestimate the occurrence of more than one headache, thus those with migraine may not report also having TTH [15]. A large proportion of non-recurrent headaches, however, are probably secondary headache disorders related to cold, fever or other diseases, or they may be very rare primary headache attacks [25]. In the present study, 77% reported having had headache, but only 29% reported recurrent headaches [12]. In one Danish study as many as 60–70% of the adolescents had infrequent TTH [26], thus one cannot exclude that subjects with sporadic primary headaches in the present study were included in the reference group and might accordingly weaken and underestimate the associations observed. The prevalence of migraine and TTH are, however, in accordance with the previous studies [27, 28]. Differences in methodology and screening question accounts for a substantial part of the various prevalence rates reported [28, 29], but a robust approach assessing comorbidity is to include only those that suffer from headaches [30].

Why are these results important? Several of the prophylactic medications used for treating migraine headache today are antihypertensive agents and the reason for their effect is not known. Nociceptive and blood pressure regulatory centers in the brain stem lie anatomically close in proximity and it has also been demonstrated that the neurons of pain regulatory centres (periaqueductal gray and rostral ventrolateral medulla) may be excited by pharmacologically induced changes in BP [31, 32].

Further research into the relationship between headache and BP may give valuable information on pathophysiological mechanisms involved in headache/migraine, general pain-modulating mechanisms and mode of action of antihypertensive drugs used for migraine prophylaxis.

## Appendix: headache description used in headache interviews

*Alternative 1* (*migraine*) This headache often comes in attacks, the pain is moderate or severe, and may start on one side of the head. The headaches are pounding or pulsating, and I prefer not to move my head. I am often nauseated and may vomit. When I have this headache light or sounds bother me.

*Alternative 2* (*tension-type headache*) This headache may start gradually and the pain is mild or moderate. The pain is usually located in the whole head, or in the neck. It feels tightening or pressing (like a band around the head). I usually have no nausea or vomiting. When I have this headache I can normally keep on with my schoolwork, homework and normal physical activity.

*Alternative 3* (*non-classifiable headache*) Neither alternative 1 or 2 describes my headache.
